# Terahertz radiation driven by two-color laser pulses at near-relativistic intensities: Competition between photoionization and wakefield effects

**DOI:** 10.1038/srep26743

**Published:** 2016-06-03

**Authors:** P. González de Alaiza Martínez, X. Davoine, A. Debayle, L. Gremillet, L. Bergé

**Affiliations:** 1 CEA-DAM, DIF, F-91297 Arpajon, France

## Abstract

We numerically investigate terahertz (THz) pulse generation by linearly-polarized, two-color femtosecond laser pulses in highly-ionized argon. Major processes consist of tunneling photoionization and ponderomotive forces associated with transverse and longitudinal field excitations. By means of two-dimensional particle-in-cell (PIC) simulations, we reveal the importance of photocurrent mechanisms besides transverse and longitudinal plasma waves for laser intensities >10^15^ W/cm^2^. We demonstrate the following. (i) With two-color pulses, photoionization prevails in the generation of GV/m THz fields up to 10^17^ W/cm^2^ laser intensities and suddenly loses efficiency near the relativistic threshold, as the outermost electron shell of ionized Ar atoms has been fully depleted. (ii) PIC results can be explained by a one-dimensional Maxwell-fluid model and its semi-analytical solutions, offering the first unified description of the main THz sources created in plasmas. (iii) The THz power emitted outside the plasma channel mostly originates from the transverse currents.

Terahertz (THz) radiation generated by ultrashort laser pulses has nowadays become an active topic of research due to many promising applications in security screening, cryptography, material sciences, medical imaging and remote detection of drugs and explosives^1^,^2^,^3^,^4^,^5^. Because these applications often request to probe unknown materials over long distances while overcoming absorption by atmospheric water molecules, there is a need for more and more intense THz signals with spectra tunable to the desired application. Several techniques exist to supply THz radiation, from conventional antennas to photoconductive switches, quantum cascade lasers, or by optical rectification in asymmetric crystals^6^,^7^,^8^,^9^,^10^. Most of them, based on solid emitters, appear to be limited by damage thresholds or by the narrowness of the emitted spectra. An alternative has been explored for more than ten years, consisting in mixing two different frequencies (colors) of intense femtosecond laser pulses inside a plasma spot that serves as a frequency down-converter in gases^11^,^12^,^13^. The resulting frequencies belong to the THz range. They can even be generated remotely in air through laser filaments^14^,^15^,^16^,^17^,^18^,^19^,^20^,^21^,^22^, produced by the interplay between the Kerr response of bound electrons and first photoionization events taking place at intensities less than 10^15^ W/cm^2^ in weakly ionized gases. Ultrabroad bandwidths up to 200 THz have also been measured in two-color gas plasma experiments^23^, bridging quasi-continuously the THz and infrared regions of the electromagnetic spectrum.

Laser-driven THz generation is, however, not limited to nonlinear optics and is now emerging as a recurrent topic in relativistic laser-plasma interaction, for instance from solid targets irradiated at intensities >10^19^ W/cm^2^ 24,25,26. In between these two working regimes lies a broad range of laser intensities, namely, 10^15^ ≤ *IL* < 10^18^ W/cm^2^, for which a plasma formed by laser-gas interaction usually involves high ionization levels and plasma waves produced in the wake of the laser field. So far, a number of papers have addressed THz wave generation by single-color pulses in classical plasma regimes^27^,^28^,^29^,^30^; some of them even examined the influence of external magnetic fields resulting in higher conversion efficiencies through three-wave parametric decay^31^. Much fewer have been devoted to two-color pulses^32^,^33^,^34^,^35^. With two colors, a well-known scenario for THz pulse generation is the photocurrent mechanism, following which ionization events in the tunneling regime accumulated along asymmetric pulse profiles produce a low-frequency current responsible for THz wave emission^36^. The robustness of this so-called photocurrent scenario at intensities above 10^15^ W/cm^2^ is, nonetheless, an open issue. Indeed, alternative key players may be longitudinal and transverse plasma waves promoted by the laser-induced ponderomotive forces^28^,^37^ or ionization-induced plasma current oscillations^38^,^39^. A pure one-dimensional (1D) configuration without ponderomotive effects was investigated by some of the present authors^35^, but a multidimensional analysis is necessary to discriminate between all the different THz sources.

In this paper, two-dimensional (2D) particle-in-cell (PIC) simulations display evidence that the photocurrent mechanism is mostly responsible for THz pulse generation at laser intensities above 10^16^ W/cm^2^ and produces GV/m field strengths in argon. However, when the relativistic intensity threshold is approached (*IL* → 10^18^ W/cm^2^), single-color and two-color pulse schemes offer similar field strengths. Close to the relativistic limit, the conversion efficiency saturates in Ar because the charge number Z^*^ = 8 corresponds to a stable electronic configuration^35^ that turns off the photocurrent mechanism. By increasing the laser intensity from 10^15^ to 3 × 10^17^ W/cm^2^, we reveal the enhanced action of longitudinal plasma waves inside the plasma channel, as the ion charge number is increased in the interval 2 ≤ Z^*^ ≤ 8. There, photoionization and longitudinal ponderomotive force act as the most efficient THz converters, whose characteristic properties are supported by a one-dimensional (1D) model unifying the THz source terms. Nevertheless, the longitudinal electrostatic fields created inside the plasma barely contribute to the outward emission of the electromagnetic fields. The transverse ponderomotive effects, instead, sustain the THz field radiated away from the plasma. As a matter of fact, we show that the magnetic field component parallel to the laser polarization axis does provide a reliable measurement of THz emissions by plasma ponderomotive forces, while the electric field polarized in the same direction mostly arises from photocurrent sources.

## Results

### 2D particle-in-cell simulations

For a few years there has been a wide consensus on the directivityof the energy radiated by a laser-created plasma spot^22^,^33^,^34^,^40^,^41^. THz emission by a plasma is usuallyevaluated through the flux of Poynting vector 
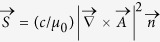
, where 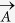 and 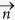 are the vector potential and propagation unit vector of the THz radiated field, respectively (*c* and *μ*_0_ are the speed of light and magnetic permeability in vacuum). Denoting by 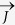 the current associated with this vector potential, 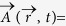

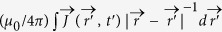
 where 
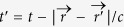
, the energy spectral density of the electromagnetic radiation per unit of solid angle Ω is computed in the far-field approximation (*r* ≫ *r*′, *c*/*ω*) as^37^


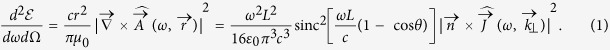


Here, 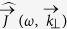 is the Fourier transform of the radiating current density expressed in the pulse retarded time *τ* ≡ *t* − *z*/*c* and in the (*x*, *y*) plane, *ω* is the pulse frequency, 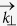 denotes its transverse wavevector, *ϕ* is the azimuthal angle while *θ* is the polar angle between 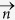 and the laser field wavevector along the propagation axis [*k*_*z*_ = (*ω*/*c*)cos *θ*]. Equation (1) holds for a current density 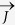 assumed to be uniform over the propagation axis *z* and whenever the laser pulse components (colors) have walk-off and phase mismatch lengths much longer than the plasma length *L*. In the following, modulations induced by mismatching between laser harmonics due to optical path difference are avoided by working with short enough plasma length (*L* ≈ 100 *μ*m). From Eq. (1) the directivity of a plasma-induced THz emission is linked to that of the current density 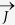 triggered by different source terms, such as photoionization or ponderomotive forces. This link is expected to provide crucial information on the nonlinearities prevailing in the far-field THz emissions, since the factor 
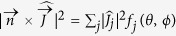
 with *j* = *x*, *y*, *z* has characteristic angular distributions 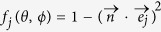, where 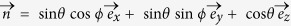. In particular, if longitudinal forces are dominant 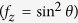, the angular distribution of the radiated power flow exhibits multiple (*N*) lobes at angular positions fixed by the relationship 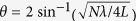, *λ* = 2*πc*/*ω* being the emission wavelength. The angular pattern here consists of two off-axis symmetric lobes (*N* = 1) and the opening angle is inversely proportional to the plasma length. When transverse forces instead prevail, the geometrical factors *f*_*x*_, *f*_*y*_ are close to unity for small angles *θ* ≪ 1 and the central lobe is filled, up to small conical deviations ≪10° 21. This feature holds except if 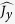 vanishes on-axis (*k*_⊥_ = 0), which will be the case for a ponderomotively driven current.

Let us recall that the applicability of equation (1) is, nevertheless, limited. Indeed, this relationship is based on a wire-antenna model that implicitly assumes no transverse variations of charge distributions. Radial ponderomotive forces do not affect the plasma channel when its transverse radius *r*_*p*_ is much smaller than the emission wavelength *λ* ≈ *c*/*ω*_p*e*_ and when THz emission proceeds from electron plasma waves with frequency *ω*_pe_. The opposite inequality, *r*_*p*_ ≫ *c*/*ω*_pe_, can also be applied to ignore transverse gradients of the laser envelope^28^. In this configuration, there is a radial component of plasma current density that induces a surface charge at the plasma-gas boundary. For a steady-state plasma, the axial (longitudinal) mode associated with *J*_*z*_ rapidly vanishes as 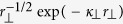 outside the plasma zone together with the radial component (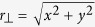, *κ*_⊥_ is a real eigenvalue). The relevant field component is the azimuthal magnetic field *B*_*θ*_ decreasing more slowly as 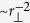 over large distances. For a non-steady plasma undergoing longitudinal ponderomotive modulations with wavenumber *κ*_*z*_, transverse modes with pure imaginary *κ*_⊥_ can be triggered and they form efficient radially outgoing waves emitted along the Cerenkov angle 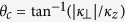 with superluminal phase velocity. Little information is, however, available on plasma geometries with finite transverse extents, for which the previous literature suggests that only the plasma-gas boundaries should emit within the plasma skin depth. To our knowledge, THz emission by photocurrents has moreover never been addressed in this context.

Below we clear up this issue and identify the main sources of THz emission at high laser intensities ≥10^15^ W/cm^2^. We performed PIC simulations using the two-dimensional version of the CALDER code (see Methods)^42^. A laser pulse polarized along the *x*-axis is propagated along a 100-*μ*m-long plasma of a 2D-geometrical (*y*, *z*) argon gas. The initial density profile is trapezoidal, with a 90-*μ*m-long plateau bordered by 5-*μ*m-long linear ramp at each side. The initial ion temperature is 1 eV and the initial neutral density, *N*_*a*_ = 2.4 × 10^17^ cm^−3^, guarantees an underdense plasma even at the highest intensities considered. We simulate a Gaussian pulse both in space and time in the form


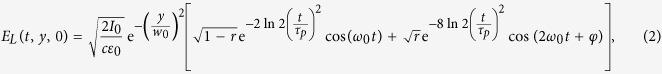


where *I*_0_ is the mean pump intensity, *r* is the relative intensity ratio of the second harmonic and *ε*_0_ = 1/*μ*_0_*c*^2^. The relative phase *φ* between the fundamental with carrier frequency *ω*_0_ and second harmonic is initially set to *π*/2. This phase offset optimizes local photocurrents^12^,^36^,^43^; however, as the relative phase evolves along propagation, the gain factor achieved with another initial phase offset would remain of similar order of magnitude (see Supplementary Information). The FWHM duration of the pump pulse centered at 1-*μ*m wavelength is *τ*_*p*_ = 35 fs and its 1/e^2^ transverse width along *y* is *w*_0_ = 20 *μ*m, allowing to form a rather thick plasma. We always consider an intensity ratio *r* of 0.1 between the fundamental pump and its second harmonic (0.5-*μ*m wavelength) when two colors are employed (*r* = 0 for a single color). Our intensity range, *I*_0_ ≤ 3 × 10^17^ W/cm^2^, corresponds to the normalized laser vector potential |*a*_*L*_| = *e*|*A*_*L*_|/*m*_*e*_*c*^2^ ≤ 0.5, where *e* and *m*_*e*_ are the electron charge and mass. The maximum values of *ω*_p*e*_, which initiate the THz spectra, are then comprised between 0.02*ω*_0_ and 0.04*ω*_0_ in argon, i.e., *ν*_pe_ ≡ *ω*_pe_/2*π* = 6–12 THz. The CALDER code encompasses all necessary plasma physics, from multiple photoionization in the tunnel regime to kinetic, collective and plasma wakefield effects, in and beyond classical plasma regimes adapted to, e.g., the physics for laser-plasma accelerators. The different radiated field components 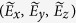 and their magnetic counterparts 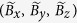, indicated with tilde symbol, are extracted from the PIC simulations. Forward radiation is only regarded in the present study and the transverse field *E*_*x*_ includes both the laser field *E*_*L*_ and the secondary (radiated) field 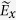. Secondary fields are polarized along their respective current densities *J*_*x*_, *J*_*y*_ and *J*_*z*_, which, by virtue of Eq. (1), should contain the spectral signature of the THz source terms at remote distances from the plasma channel. In our 2D (*y*, *z*) geometry, the relationship 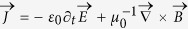 invites us to map the THz spectrum yielded by *J*_*x*_ from *E*_*x*_ in order to capture photocurrents only, and use *B*_*x*_ = ∂_*y*_*E*_*z*_ − ∂_*z*_*E*_*y*_ to isolate contributions subject to the ponderomotive forces acting in the orthogonal plane.

Although our simulation parameters are chosen for an academic purpose, they can be approached using gas-jet tailoring techniques developed for laser-wakefield accelerators. In this setting sharp downward density transitions are created through shock fronts induced either by a knife edge or by nanosecond laser pulses into a supersonic gas target^44^,^45^. A typical scheme could here employ 35-fs, 70-mJ pump pulses focused into a vacuum chamber by a *f/*10 off-axis mirror (numerical aperture ~0.02) and passing through a 0.1 mm thin *β*-barium borate (BBO) crystal. At the centre of the vacuum chamber, the nozzle delivering a supersonic Ar-gas jet would host a razor blade mounted laterally, allowing to switch a shock front transversally along the laser propagation axis and to monitor density gradients over ~10 *μ*m lengths. The forwardly emitted THz radiation would then be collected by a parabolic mirror and directed toward a pyroelectric detector equipped with a silicon filter.

**Figure 1:**
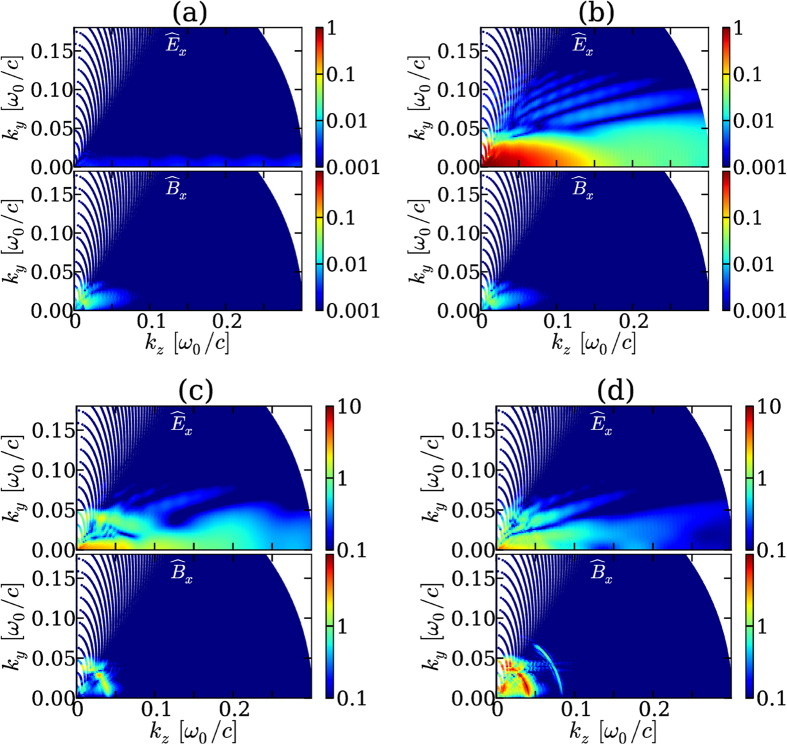
Spectral angular distribution of THz radiation (arb. units) in the (*k*_*y*_, *k*_*z*_) plane produced by *E*_*x*_ and *B*_*x*_ along the laser polarization axis in the following configurations: (**a**) Single-color pulse and (**b**) two-color pulse with mean intensity *I*_0_ of 10^15^ W/cm^2^; (**c**) two-color pulse with 5 × 10^16^ W/cm^2^ and (**d**) two-color pulse with 3 × 10^17^ W/cm^2^. The fields are recorded as a function of *t* and *y* at the given position *z* = 175 *μ*m (outside the plasma zone) and their spectra are discretized on a uniform, Cartesian mesh as a function of *ω* and *k*_*y*_. Due to the quadratic dependence of 
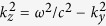
 on *ω* and *k*_*y*_, the fields are plotted on a non-uniform mesh as a function of *k*_*z*_ and *k*_*y*_. Each point in the (*k*_*z*_, *k*_*y*_) space is represented by a color dot. The overlap of the dots creates the color map; this overlap cannot be achieved for the very low frequencies *k*_*z*_ ≪ *k*_*y*_.

Figure 1 shows our principal result which, to the best of our knowledge, consists of the first spectral mapping of laser-plasma-based THz generation accounting for both photocurrents and ponderomotive forces in multidimensional geometry. From top to bottom are presented the spectra of the radiated field component 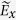 and of the transverse magnetic field 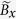. The two-color laser field is linearly polarized on the *x*-axis, along which no propagation or plasma interaction effects occur. The angular distribution in (*k*_*y*_, *k*_*z*_) of 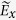 thus reflects in Fourier space THz emission by photoionization, since 
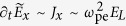
 (see equation for 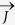 above). Here the electron plasma frequency 
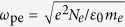
 increases with the free electron density *N*_*e*_(*t*) along fast ionization steps^36^ and reaches its maximum value beyond the laser region. Along the same direction, the polar emission map of 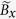 displays THz radiation from 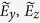, which excludes photoionization but keeps the trace of ponderomotive effects in the *y* − *z* plane.

For the main three intensity levels investigated here, (10^15^, 5 × 10^16^ and 3 × 10^17^ W/cm^2^), THz emissions forwarded beyond the plasma zone (*z* = 175 *μ*m) exhibit similar spectral signatures. Photocurrents induced by tunneling ionization are known to play a major role in THz generation from gases irradiated by two-color laser pulses at filamentation intensities ~10^14^ W/cm^2^ 19. Recently, this property was confirmed by direct measurements supported by numerical simulations of two-color filaments in air, the plasma response of which was shown to take over the Kerr nonlinearity in the conversion process, shifting the peak of the THz spectrum towards the electron plasma frequency^46^. For one order of magnitude larger intensities, photocurrents again persist as being the major mechanism in driving THz emissions, whereas single-color pulses mostly initiate THz radiation from plasma wakefields [compare Fig. 1(a) and Fig. 1(b)]. One or two-color schemes do not change the ponderomotive contributions to the THz yield. The *E*_*x*_-field spectra, peaked on-axis (*θ* = 0), decay at larger *θ* angles according to the sinc function of Eq. (1). At laser intensities high enough to promote large ion charge numbers Z^*^ = *N*_*e*_/*N*_*a*_ → 8, photoionization still competes with ponderomotive effects for emitting THz electromagnetic fields, despite the important strength of the ponderomotive sources [see Fig. 1(c)]. Close to the relativistic limit, *I*_0_ = 3 × 10^17^ W/cm^2^, the conversion efficiency due to photocurrents somewhat saturates, but goes on delivering intense THz pulses [Fig. 1(d)]. In summary, along *x*, photocurrents increase the THz yield as the pump intensity is augmented till saturation at near-relativistic intensity. THz components due to ponderomotive emitters along the orthogonal directions, by contrast, monotonically increase.

### A simplified model for *in-situ* THz emitters

Understanding the previous results requires to scan the field dynamics initiated inside the plasma channel. For simplicity, we use a reduced model discriminating THz emitters only promoted by the *x*– and *z*–polarized fields, since photocurrents generate THz pulses along the laser polarization axis and propagation aspects mainly concern the longitudinal axis. Discarding ion motions, the current density induced by the photoionized electrons is 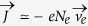, where *N*_*e*_ and 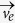 are the free electron density and velocity. The electron current obeys the following equation set^28^


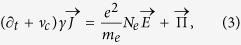



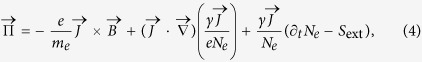


where 
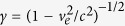
 is the relativistic factor, *ν*_*c*_ is the electron collision rate, 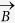 is the magnetic field associated with the total electric field 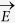 that includes both the laser field and secondary (THz) radiation. The last term of Eq. (4) originates from external drivers, such as photoionization, electron recombination or attachement. Over femtosecond time scales, only photoionization plays a role. This process is described using an Ammosov-Delone-Krainov (ADK)-based multiple ionization scheme^47^ that involves the increase in ion densities gathered within the source term *S*_ext_ = ∑_*j*_* j*∂_*t*_*N*_*j*_ (see Methods). In our selected intensity range, tunnel ionization indeed prevails as argon atoms are never fully ionized and their electron shells undergo multiple sequential ionization. The other terms refer to the radiation pressure and ponderomotive forces, which may be evaluated using envelope substitutions, i.e.,


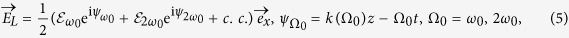


where 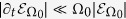. Combining Eqs (3) and (4) with Maxwell equations provides the propagation equation for the overall electric field 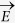 and yields the full three-dimensional Maxwell-fluid model. This is reduced to a one-dimensional (*z*-propagating) model by discarding the diffraction operators (∂_*x*_ = ∂_*y*_ = 0) in the transverse plane together with chromatic dispersion. Since our intensity range remains sub-relativistic, we moreover omit variations of the relativistic factor and 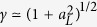 is viewed as a constant. In this regime the nonlinear source term 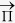 is evaluated on the laser field propagating forward with the sole variable (*z* − *ct*). Ignoring terms in 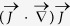 at leading order and assuming 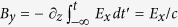 for *B*_*y*_ travelling like the laser pulse, our model equations for the transverse and axial fields are:


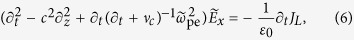



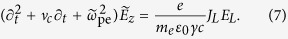


They both involve the current density computed on the laser field, 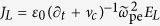, and the modified electron plasma frequency


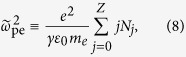


that incorporates the fast growth of ion densities *N*_*j*_ over attosecond ionization time scales. At high intensities, temporal modulations of *N*_*e*_(*t*) due to plasma wakefield is described by the relationship 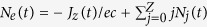.

The transverse field contains two contributions to THz emission: (i) the coupling between *N*_*e*_(*t*)∼∑ _*j*_* jN*_*j*_(*t*) and the high-frequency field *E*_*L*_(*t*) creating photocurrents through the laser current density 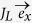 and (ii) plasma current oscillations developing over long times^36^,^39^. With two colors, the product between the fast variations of *N*_*e*_(*t*) and those of *E*_*L*_(*t*) acts as an efficient converter to low frequencies and promotes slow current components associated with THz generation in the beam head^13^. By comparison, the longitudinal field equation (7) describes THz emission from plasma oscillations that develop over picosecond time scales after the laser field has interacted with the gas. Collisions are weak (8.83 × 10^−5^ ≤ *ν*_*c*_ ≤ 5 × 10^−3^ fs^−1^, see Methods) and THz emissions due to longitudinal fields are mainly triggered by ponderomotive excitations^28^,^37^.

**Figure 2:**
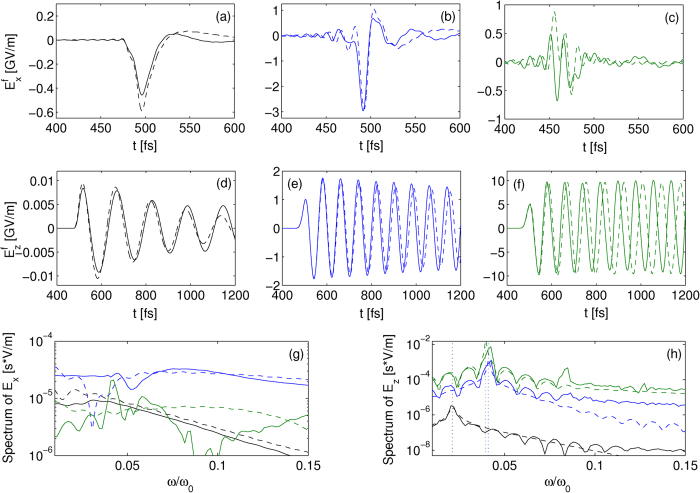
Comparison between 2D PIC results (solid curves) and semi-analytical solutions of the 1D model (dashed curves) for the on-axis electric fields (top row) 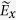 and (middle row) 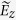 emitted by two-color pulses inside the plasma channel at *z* = 50 *μ*m for the mean pump intensity (**a,d**) *I*_0_ = 10^15^ W/cm^2^ (black curves), (**b,e**) *I*_0_ = 5 × 10^16^ W/cm^2^ (blue curves), and (**c,f**) *I*_0_ = 3 × 10^17^ W/cm^2^ (green curves). (Bottom row) On-axis spectra of the radiated fields (**g**) 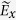 and (**h**) 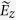 with same color plotstyles. The vertical dotted lines indicate values of the plasma frequency.

Figure 2(a–f) proceed with a direct comparison between the on-axis transverse (*x*) and axial (*z*) components of the electromagnetic field emitted in a 90-THz wide frequency window (*ω*/*ω*_0_ < 0.3) at the middle of the plasma channel, *z* = 50 *μ*m. Black, blue and green curves in solid lines refer to CALDER computations for increasing intensities; dashed curves plot the corresponding solutions to Eqs (6) and (7) with the same color plotstyle. The latter equation for 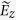 is solved as an ordinary differential equation by a classical Runge-Kutta method; the former for 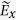 is a linear transport equation solved through standard upwind finite difference schemes. Equation (6) indicates that the transverse radiated field strength intrinsically depends on the longitudinal gradients. Compared with antecedent studies assuming *a priori* 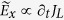
36, Eq. (6) directly provides the correct field strength with no need of geometrical factor^48^. The dominant part of its solution is a single-cycle pulse due to photoionization, as plasma current oscillations only form a residual tail modulated at plasma frequency [Fig. 2(a–c)]. The axial field is connected with plasma wave oscillations, where the plasma frequency increases like 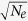, i.e., with the ionization state Z^*^ when the laser intensity is increased [Fig. 2(d–f)]. For each intensity value, Fig. 2 displays evidence of a very good agreement between 2D PIC computations and our 1D model. Small discrepancies occur in the THz pulse profiles at near-relativistic intensity. These are linked to two-dimensional effects (e.g., transverse diffraction), small deviations from the assumption of constant *γ*, and the non-negligible influence of electron-electron collisions. They, however, preserve an accurate estimate of the THz field strength. Our 1D model provides us with an analytical evaluation for the transverse emitted field (see Methods):


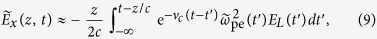


while the axial field recovers the standard form (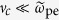)


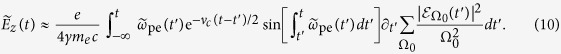


Maximum achievable field strengths are 3 GV/m for the transverse field produced at 5 × 10^16^ W/cm^2^ and 10 GV/m for the longitudinal field emitted at 3 × 10^17^ W/cm^2^ inside the plasma. These are record values reported in this context.

Figure 2(g,h) detail the spectral dynamics inferred from our 1D model for increasing intensities. Again we let the pulse propagate over 100 *μ*m of plasma, then filter out the emitted fields at *z* = 50 *μ*m. The dotted vertical lines locate the plasma frequency 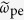 achieved in the wake of the laser field and expressed in *ω*_0_ units. In Fig. 2(h) the spectrum of the longitudinal field remains highly peaked around the plasma frequency, as justified by its direct dependency on 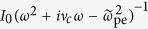, which can easily be sorted out from Eq. (7). By contrast, the spectrum of the transverse field increases in amplitude and toward “long” wavelengths with the laser intensity, before saturating at 3 × 10^17^ W/cm^2^ [Fig. 2(g)]. Its analytical expression established by taking the Fourier transform of Eq. (6) reads as


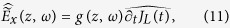


assuming a constant plasma frequency, where the *z*-dependent function *g*(*z*,*ω*) can be found detailed in the Methods section. With a constant *g* one retrieves the well-know local current relationship 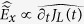
36. Initiated around the plasma frequency (0.02 < *ω*_pe_/*ω*_0_ ≤ 0.04), the transverse THz spectrum develops a broad extent comparable with the patterns of Fig. 1. The overall agreement between PIC simulations and our 1D solutions is good, except in the very low-frequency limit.

**Figure 3:**
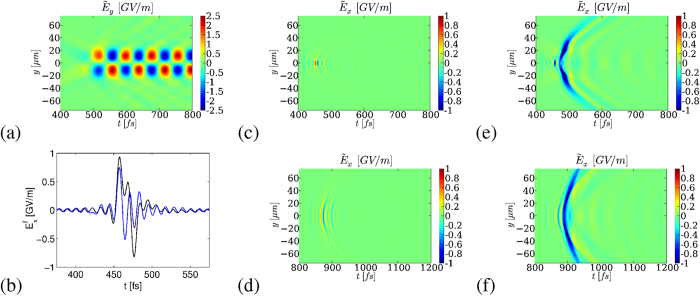
(**a**) 2D PIC spatio-temporal map of 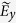 triggered by two-color pulses for *I*_0_ = 3 × 10^17^ W/cm^2^. (**b**) 2D PIC simulation (blue curve) and 1D semi-analytical solution (black curve) of the on-axis *x*-polarized radiated field at *z* = 50 *μ*m for a single color with the same intensity. Spatio-temporal maps 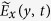 for (**c**) a single-color pulse and (**e**) a two-color pulse at the same distance and intensity. (**d,f**) Same patterns at remote distance *z* = 175 *μ*m outside the plasma channel. The color bars of (**c–f**) are cut at ±1 GV/m for better visibility of the emitted waves. Note the change of scale in time.

Figure 3(a) confirms that, at the highest intensity *I*_0_ = 3 × 10^17^ W/cm^2^, the *y*-polarized fields induced by transverse ponderomotive forces generate *in-situ* off-axis THz components 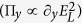 being weaker than their longitudinal counterpart [compare with Fig. 2(f)]. This justifies a posteriori our 1D model. At the same intensity level and for one color, the on-axis CALDER field 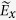 and its semi-analytical evaluation are shown in Fig. 3(b), evidencing that our semi-analytical model again provides reliable approximations at near-relativistic intensities for a single-color pulse, apart from the aforementioned discrepancies. By comparison of this subplot with Fig. 2(c), we can observe that both the single- and two-color pulse schemes supply analogous on-axis *x*-field strengths (~GV/m). The maximum THz field amplitudes delivered by tunnel ionization being located on-axis, one infers a clear saturation in the conversion efficiency of the two-color scheme near the relativistic intensity threshold. The reason of this saturation is that, at intensities close to 10^18^ W/cm^2^, Ar atoms have their outermost electron shell empty (Z^*^ = 8); so the remaining ion is shaped into a stable Ne-like atom configuration. This property manifests itself by a long plateau in the curve Z^*^ versus *I*_0_^35^, signaling the hardness to further ionize the ion. Approaching Z^*^ = 9 with 1.4 × 10^18^ W/cm^2^ intensities re-activates the two-color pulse efficiency by the delivery of 7.6 GV/m THz transverse fields (not shown), which, however, remains comparable with the present performances. This explains why the ionization process loses efficiency near relativistic intensities. The same saturation phenomenon can be expected in other gases, for instance helium, once the available two electrons have been ionized at similar intensities. It is worth noticing that although photocurrents cannot produce stronger THz fields, they can supply more energetic THz pulses as the volume of the secondary radiation is broader along the *y*-axis for two colors [compare Fig. 3(c) and Fig. 3(e)]. Figure 3(d,f) show that the field values ~1 GV/m achieved inside the plasma are preserved at remote distances.

### Leaking secondary fields

**Figure 4:**
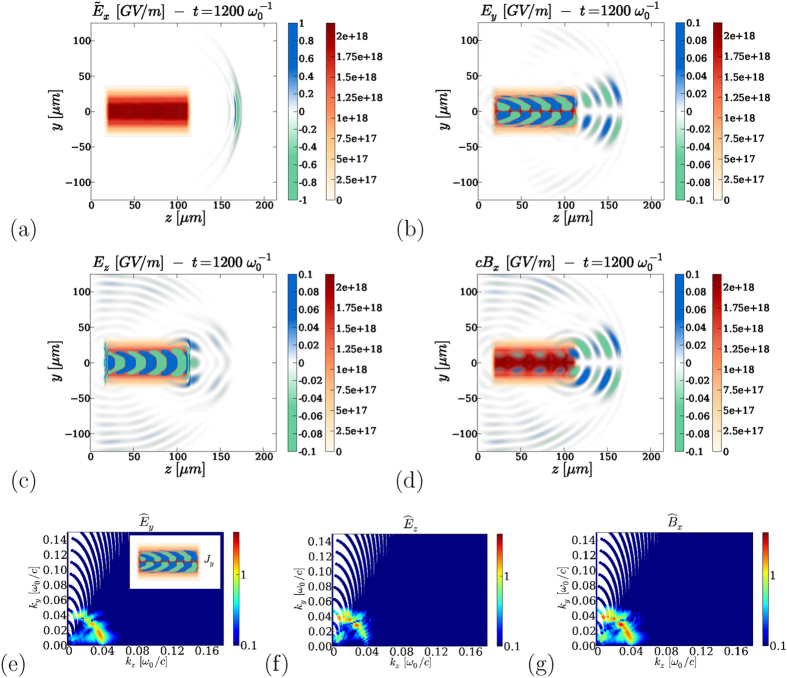
Two-color pulses for *I*_0_ = 5 × 10^16^ W/cm^2^. Top: (*y*, *z*) mapping of the fields emitted inside and outside the plasma channel at time 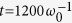. The left-hand side blue/green color bar indicates the field strength value in GV/m; the right-hand side red color bar indicates the electron density in cm^−3^. (**a**) 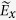 (the laser field is filtered out), (**b**) *E*_*y*_, (**c**) *E*_*z*_ and (**d**) *cB*_*x*_. Bottom: Angular spectral distribution (arb. units) of (**e**) 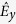, (**f**) 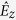, and (**g**) 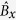 at *z* = 175 *μ*m. In (**e**) the inset represents the *y*-component of the current density generated inside the plasma.

While the secondary transverse and longitudinal fields contain the THz signature of their respective nonlinear sources, it is not guaranteed that outside the plasma channel these components preserve their field strength - thus the transmitted THz power - all the way to the detector. Figure 4 answers this point by detailing the (*y*, *z*) maps of the electric field and *x*-magnetic components inside the plasma zone and transmitted in vacuum, once the pulse has propagated along the whole gas length (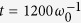). The free electron density (red color bar) and the field amplitude level (blue/green color bar) are specified. It is clearly seen that the THz field 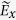 created through photoionization keeps an amplitude ~GV/m comparable with the one produced inside the plasma tube, as already reported above. By contrast, 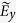 decreases by a factor ~2.5 and 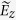 vanishes rapidly. Along *y*, transverse ponderomotive forces generate obliquely-propagating THz pulses. This behavior can be understood from the ponderomotive source term 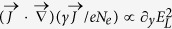 computed on the laser field Eq. (2), which is zero at *y* = 0 and maximum near *y* = 10 *μ*m. For comparison, the longitudinal field is maximum at center (*y* = 0), but its amplitude rapidly falls down outside the plasma channel. Amongst these two players, the transverse ponderomotive force hence conveys the highest THz field contribution, unlike the longitudinal field that becomes unable to transmit the THz pulse. As shown by Fig. 4(d), the peaks of the magnetic field 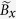 outside the plasma are mainly those of the 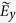 component. Inside the plasma region, 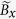 arises from the current components in the transverse gradients of the plasma profile. Spectrally, 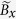 keeps the signature of the transverse and longitudinal fields created inside the plasma channel, in such a way that it consists in the direct sum of 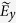 and 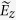 conical emissions. In this respect, Fig. 4(e–g) indeed display evidence that the longitudinal field spectrum 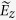 is non-zero at an angle larger than that of 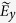, so that 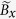 simply superimposes both contributions. In connection with Eq. (1), the inset of Fig. 4(e) reveals that *J*_*y*_ is an odd function of *y* and has no on-axis spectral component 
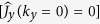
. The transverse ponderomotive currents, therefore, emit oblique THz waves, which was never reported before.

**Figure 5:**
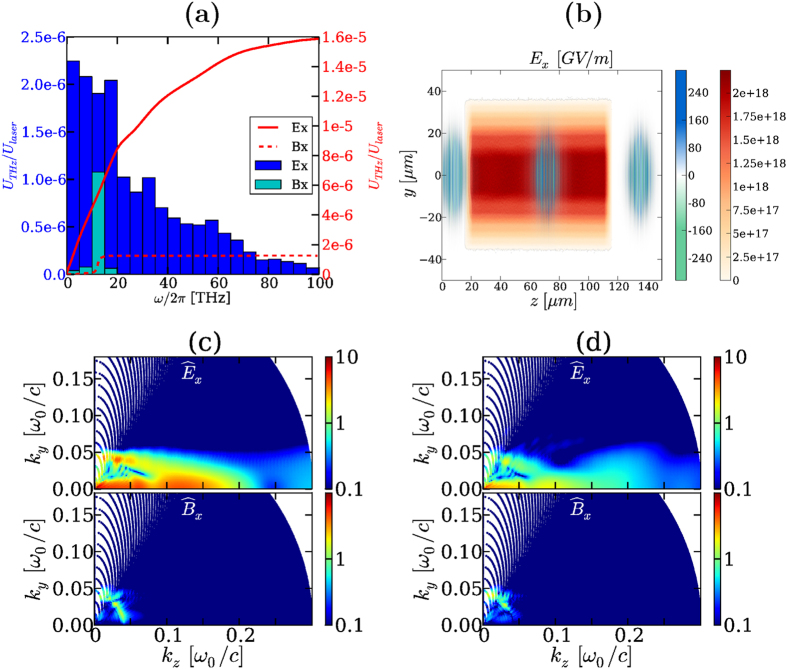
(**a**) THz energy distribution *U*_THz_ normalized to laser energy versus the electromagnetic frequency *ν* ≡ *ω*/2*π* at *z* = 175 *μ*m in the two-color case with *I*_0_ = 5 × 10^16^ W/cm^2^ from the ponderomotive forces (*B*_*x*_) and photocurrents (*E*_*x*_). The solid line represents the cumulative distribution function in frequency that integrates the THz energy yields over the antecedent frequency domains. (**b**) Spatial map of the laser field *E*_*x*_ at three different propagation distances along and outside the plasma channel (the electron density is plotted in red). (**c,d**) Influence on the angular far-field spectra (arb. units) of the laser pulse duration and longitudinal plasma profile for the same intensity and (**c**) a pump FWHM of 17.5 fs (second harmonic length remains unchanged) and a higher initial ion density level *N*_*a*_ ≈ 5 × 10^17^ cm^−3^ at *z* = 175 *μ*m (*φ* = 0); (**d**) a longer plasma gradient [50 *μ*m instead of 5 *μ*m in the rear part of the plasma profile, which still extends over 100 *μ*m; the laser parameters are unchanged compared with Fig. 1(c)]. The *x*-field spectrum radiated by photocurrents built from fewer optical cycles is broader and more intense.

To end with, we present in Fig. 5(a) the energy converted to the THz and infrared domains over the 100-*μ*m long plasma channel for the same laser characteristics as those of Fig. 4. We can see that the energy radiated outside the plasma region (*z* = 175 *μ*m) is located around the plasma frequency (~10 THz) due to wakefield and plasma wave emitters (*B*_*x*_). Yet most of this energy results from photoionization (*E*_*x*_) and accumulates over a broader frequency range. This property is reflected by the solid red line, plotting the cumulative distribution function in frequency (expressed in THz for better convenience). We moreover evaluated the optical energy lost through photoionization, backscattering and electron acceleration. These correspond to 0.06% of the input laser energy and amount to a conversion efficiency of about 2.6% over all the consumed laser energy in our frequency window. This value is weak, i.e., the plasma-to-THz conversion scheme is not optimal, but it is achieved over 100 *μ*m of an underdense plasma only. For completeness, Fig. 5(b) details the evolution of the spatial structure of the laser field along the propagation range exceeding the plasma zone. This map clearly shows that over short propagation ranges the laser pulse profile remains preserved, which is consistent with the small energy losses.

The previous behaviors, of course, depend on the number of optical cycles contributing to THz pulse generation, on the initial gas atomic density and on the laser and plasma geometry. However, they should be generic. Figure 5(c,d) confirm this expectation by showing 2D PIC simulations of a two-color pulse whose fundamental has 17.5 fs FWHM duration and interacts with a twice higher initial density of atoms [Fig. 5(a)] or with a plasma profile having a longer gradient length [Fig. 5(b)]. The spectral pattern delivered by 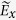 exhibits a broader extent and higher amplitude, as fewer optical cycles render the pump profile more asymmetric^39^, and there is a decrease in the ponderomotive spectral signal of 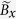 with longer density gradients^26^. Apart from these modifications, the THz spectra present generic features and we can anticipate that in a three-dimensional geometry the field distributions should remain close to the present ones, including solely an additional ponderomotive component along the *x*-axis.

## Discussion

We reported PIC simulation results on the interaction of two-color laser Gaussian beams with an underdense plasma generating THz pulses. Our objective was to discriminate THz emissions promoted by photocurrents along the laser polarization axis from those produced by plasma wave oscillations that develop at high intensities. These results have been corroborated by a robust, semi-analytical 1D model. This model can be faithfully used for a broad range of laser intensities to predict THz fields occurring inside plasmas through photoionization and longitudinal wakefields in various experimental setups. We showed that, in the range of laser intensities between 10^15^ W/cm^2^ and 3 × 10^17^ W/cm^2^ covering the domain of classical laser-driven plasma physics, THz pulse generation proceeds from both photoionization and ponderomotive forces. While the photocurrent mechanism prevails at intensities of 5 × 10^16^ W/cm^2^ in argon, the resulting THz field strength saturates when the relativistic limit *I*_0_ → 10^18^ W/cm^2^ is approached. This saturation stems from the fact that all outermost electrons of the valence shell have been ionized, leaving the ion in a relatively stable atomic state, unless resorting to much higher relativistic intensities. Finally, we demonstrated that the longitudinal fields alone rapidly decay away from the plasma and cannot transmit significant THz power remotely. It turns out that in multidimensional configurations the fields produced by transverse ponderomotive forces prevail outside the plasma zone over their longitudinal counterparts. Therefore, the radiated THz magnetic field along the laser polarization axis conveys the most relevant information on the plasma ponderomotive emitters.

## Methods

### 2D PIC Simulations

Two-dimensional particle-in-cell simulations have been performed using the parallelized CALDER code^42^, which solves the coupled set of Vlasov and Maxwell equations. This kinetic description, which entails a very large computational load, is required for the strongly nonequilibrium physics of intense laser-matter interaction. The Vlasov equation is solved by discretizing the plasma as a collection of charged “macro-particles”. Maxwell equations are discretized on a regular, fixed and staggered Cartesian mesh according to the time-explicit Yee method. In our simulations, the resolution in space and time is Δ*t* = 0.079 fs, Δ*y* = 0.48 *μ*m, Δ*z* = 0.024 *μ*m and the spectral step is Δ*ν* = 0.47 THz. The CALDER code includes Ammosov-Delone-Krainov (ADK)-based strong-field ionization modules^49^ and Coulomb binary collisions^50^. Concerning the latter, we performed several tests on the impact of electron-electron and electron-ion collisions in the THz conversion efficiency and spectral patterns. We found a limited action of electron-electron collisions for intensities less than 10^17^ W/cm^2^. Instead, electron-ion collisions dominate over long times and mainly condition the slow exponential damping of the longitudinal plasma waves. At larger, near-relativistic intensities, electron-electron collisions can, however, take over when the electrons acquire high drift velocities. For each simulation set, the collisional rate changes with the plasma characteristics and input laser parameters. Our code does not describe electron-neutral collisions, which is a valid approximation for sufficiently ionized plasmas. With free electron densities about ≈10^18^ cm^−3^ levels, electron-ion collisions always persist with an effective rate *ν*_*c*_, which varies with the input laser intensity and the achieved electron density as *ν*_*c*_ [fs^−1^] ~7 × 10^−6^ *N*_*e*_[cm^−3^]
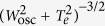
, where *W*_osc_ ~ *I*_0_ is the electron oscillation energy in the laser field and *T*_*e*_ denotes the electron temperature in eV^34^. Collisions thus decrease at increasing laser energy, up to the variations in the electron temperature that can reach ~0.1 keV in our intensity range. 2D PIC simulations indicate an effective electron-ion collision time of ~200 fs at 10^15^ W/cm^2^ intensity, being characteristic of weakly ionized gases^13^, of about 1 ps at 5 × 10^16^ W/cm^2^ intensity, and a collision time larger than 10 ps at 3 × 10^17^ W/cm^2^ intensity. All these values satisfy 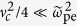.

The next step consists in comparing 2D PIC simulations with a simpler 1D model discarding transverse ponderomotive and diffraction effects in the plasma. Semi-analytical solutions are then extracted from this 1D model, which enables us to approximate with accuracy the transverse and axial THz emissions for a broad intensity range.

### Propagation equations

Working with high laser intensities and strong plasma excitations, we assume a minor role from bound electrons and discard all optical effects, such as Kerr self-focusing and chromatic dispersion. The laser pulse travels with group velocity equal to *c* and the plasma dynamics are described by the cold plasma fluid equation^28^,^51^


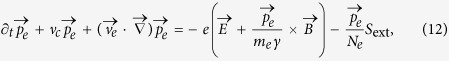


where 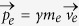 is the electron momentum [
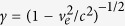
], 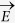 and 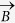 are the electric and magnetic fields, and *N*_*e*_ denotes the free electron density. 
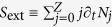
 refers to the photoionization source where *N*_*j*_ are the ion densities evolving as


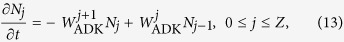


where 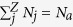, *N*_*a*_ and *Z* being the initial gas density and the atomic number, respectively^35^. The ADK-based ionization rate of complex atoms in the tunneling regime reads^47^,^49^,^52^


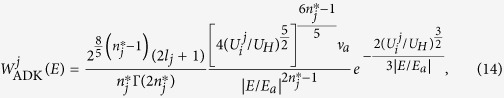


where 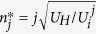 and *l*_*j*_ are the effective principal and orbital quantum numbers, 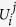 is the ionization energy of the *j*th electron of argon atoms^53^, *ν*_*a*_ = 4.13 × 10^16^ Hz, *E*_*a*_ = 5.14 × 10^11^ V/m (Hartree atomic units) and *U*_*H*_ = 13.6 eV is the ionization potential of hydrogen. The electron density *N*_*e*_ evolves as


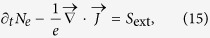


where the current density, neglecting ion motions, is given by 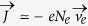. Combining Eqs (12) and (15) straightforwardly yields Eqs (3) and (4). By using Maxwell equation 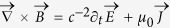, these provide the propagation equation for 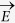:


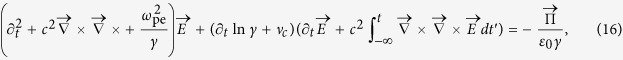


where 
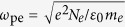
 is the standard electron plasma frequency. We here omit loss currents due to plasma absorption, which are small in underdense plasmas. The electric field 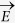 contains both the laser electric field polarized along the *x*-axis and the radiated field components.

Our one-dimensional model derives from Eq. (16). We neglect the diffraction operators (∂_*x*_ = ∂_*y*_ = 0), yielding


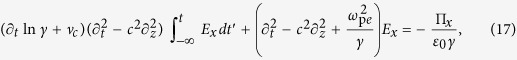



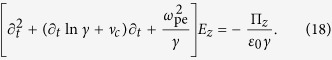


Next we evaluate the source terms 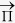 of Eq. (4) on the laser field and thus need to determine the associated current density. Its expression proceeds from equation (3), for which an underlying hypothesis is always 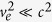 for massive (electron) particles. In these conditions, the ponderomotive terms such as 
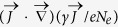
 can be assumed of second order, so that


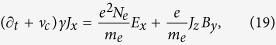



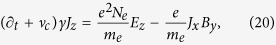


where 
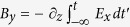
. *E*_*z*_ is the longitudinal field produced by the laser field and obeying Gauss law


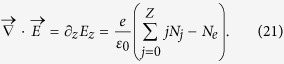


Further approximations are requested to achieve a tractable theoretical model. First, we limit our intensity range to marginally-relativistic intensities, i.e., the normalized laser vector potential satisfies *a*_*L*_ ≤ 0.5, corresponding to laser intensities *I*_0_ ≤ 3 × 10^17^ W/cm^2^. This amounts to considering relativistic factors 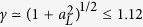. So, *γ* is valued to only about 10% above unity and we assume it constant (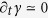). Second, the last term of Eq. (4) vanishes when it is computed in the laser region, since the photoionization model supposes ∂_*t*_*N*_*e*_ = *S*_ext_ for electrons ionized with zero velocity at the electric field maxima^36^. Because ∂_*x*_*J*_*x*_ = 0, transverse ponderomotive effects are ignored and thus


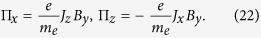


By virtue of Eq. (15), since *J*_*z*_ is initiated by the laser field, function of (*z* − *ct*), the electron density *N*_*e*_(*t*) expresses as


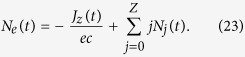


### The transverse field

To find out the transverse field, we transform Eq. (17) by applying ∂_*t*_ ln *γ* = 0 together with Eq. (22) and 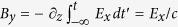 for *B*_*y*_ travelling like the laser pulse, from which


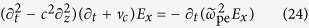


easily follows and is further numerically solved. Using 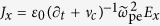 and splitting 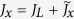 on the expansion 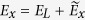, the previous equation produces Eq. (6).

We re-express this equation in the variables *τ* = *t* − *z*/*c*, *η* = *ct*. By assuming a constant effective plasma frequency 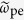 in its left-hand side and ignoring the *η*-derivatives in the weak collisional term ≈(1 + *ν*_*c*_/∂_*τ*_)^−1^, a direct Fourier transform gives the spectrum of 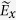, Eq. (11), where


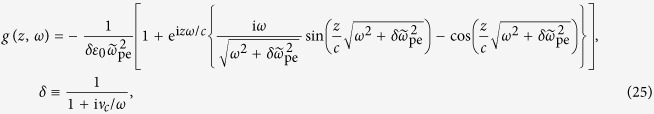


involves the propagation variable *z* for which the pulse center is *τ* = 0 in the group-velocity frame. 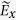 can then be extracted by inverse Fourier transform.

Alternatively, solutions to Eq. (24) can be sought as 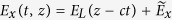 inside two distinct domains^39^: the so-called “beam head” domain, in which the radiated field 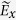 is small in front of the laser field, 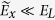 for *τ* < *τ*_*p*_. In that case the right-hand side of Eq. (24) reduces to 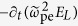. By solving the resulting equation in the co-moving frame travelling with the laser pulse group velocity *τ* = *t* − *z*/*c* and *ξ* = *z* along the characteristics (*τ* − *τ*_0_)/2 = (*ξ*_0_ − *ξ*)/*c*, one extracts Eq. (9).

Behind the beam head, for long times *τ* ≫ *τ*_*p*_, 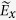 is computed from 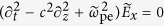 in the limit of weak collisions. By means of Laplace transforms, the solution 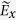 consists of residual plasma oscillations shaped by the Bessel function of the first kind, which forces the field to decay like 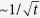 over long times (see Debayle *et al.*^39^ for technical details).

### The longitudinal field

Since *E*_*x*_ is dominated by the laser field *E*_*L*_, *B*_*y*_ reduces to *B*_*L*_ = *E*_*L*_/*c* in Π_*x*_, *J*_*x*_ ≈ *J*_*L*_ and the longitudinal radiated field 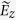 obeys


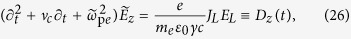


which is nothing else but Eq. (7). Here we approximate *N*_*e*_ by the sum 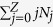 at first order, i.e., the effective plasma frequency 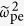 is not an implicit function of 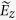 and it is defined by Eq. (8). Equation (26) has thus the direct solution:


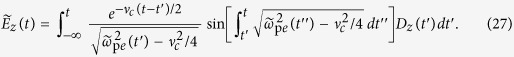


When the right-hand side term of Eq. (18) is evaluated through the slowly-varying envelope function (5), the corresponding magnetic field and current density admit the following decompositions:


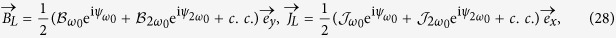


where, for Ω_0_ = *ω*_0_ or 2*ω*_0_,


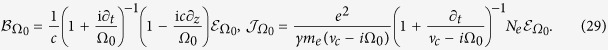


Applying the slowly-varying envelope approximation ∂_*t*_ ≪ Ω_0_, we readily find


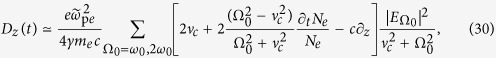


and Eq. (27) reproduces Eq. (10) using 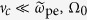 and 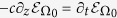. Similar expressions were earlier derived by Sprangle *et al.* and D’Amico *et al.* for a single wave (Ω_0_ = *ω*_0_) in the non-relativistic limit *γ* = 1^28^,^37^. Besides *γ* ≠ 1, the three differences with these former works are the following.

- In Eqs (26) and (27), the product of the rapid variations of *N*_*e*_(*t*) in 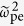 through photoionization in the beam head [∂_*t*_*N*_*e*_(*t*)~*S*_ext_] yields non-zero field contributions in the THz domain.
- We account for wakefield plasma oscillations linked to a non-zero longitudinal field (*J*_*z*_ ≠ 0) increasing from high enough laser intensities.
- Our model equations apply to two-color pulses and can easily be extended to more colors.

The characteristic behaviors of the emitted fields and additional validations of Eqs (6) and (7) compared to solutions of Eqs (17) and (18) are detailed in the Supplementary Information. They confirm that the one-dimensional model provides us with a good tool for interpreting 2D PIC simulations of laser-driven THz radiations in the plasma region.

## Additional Information

**How to cite this article**: González de Alaiza Martínez, P. *et al.* Terahertz radiation driven by two-color laser pulses at near-relativistic intensities: Competition between photoionization and wakefield effects. *Sci. Rep.* **6**, 26743; doi: 10.1038/srep26743 (2016).

## Supplementary Material

Supplementary Information
