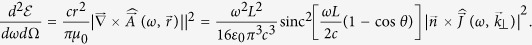# Corrigendum: Terahertz radiation driven by two-color laser pulses at near-relativistic intensities: Competition between photoionization and wakefield effects

**DOI:** 10.1038/srep33941

**Published:** 2016-09-28

**Authors:** P. González de Alaiza Martínez, X. Davoine, A. Debayle, L. Gremillet, L. Bergé

Scientific Reports 6: Article number: 2674310.1038/srep26743; published online: 06 03 2016; updated: 09 28 2016

This Article contains a typographical error in Equation 1,


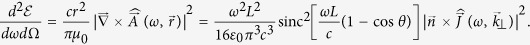


should read: