# Supplementary material for: Terahertz radiation driven by two-color laser pulses at near-relativistic intensities: Competition between photoionization and wakefield effects
Source: Sci Rep. 2016 Jun 3;6:26743. doi: 10.1038/srep26743 (PMC4891721; doi:10.1038/srep26743)
Supplement: Supplementary Information [file srep26743-s1.pdf]

Supplementary Information

Terahertz radiation by two-color laser pulses at near-relativistic intensities: Competition between photoionization and wakefield effects

P. González de Alaiza Martínez[1,*], X. Davoine[1], A. Debayle[1], L. Gremillet[1], and L. Bergé[1]

[1]CEA-DAM, DIF, F-91297 Arpajon, France
[*]pedro.gonzalesdealaiza@cea.fr

Numerical solutions of the one-dimensional model

This Supplementary Material aims at testing the field dynamics of our 1D model and compares its solutions with those of the original equations (17) and (18). Here and below, the equation numbers refer to the equations of the main manuscript.

To capture the characteristic signatures of the emitted fields, we integrate Eqs (6) and (7) using the initial two-color laser pulse Eq. (2) along $y = 0$ with $\tau_p = 35$ fs, $\omega_0 = 1.88$ fs^{-1} (carrier wavelength of 1 μm) interacting with argon at low pressure ($N_a = 2.4 \times 10^{17}$ cm^{-3}). The electron collision rate is here fixed to $\nu_c = 1.3$ ps^{-1}. Once the differential equations are solved for one ($r = 0$) and two colors ($r = 0.1$), we plot their respective solutions filtered in the THz frequency window $\nu < 90$ THz ($\omega/\omega_0 < 0.3$) and show their associated THz spectra computed in the middle of the plasma channel ($z = 50\,\mu$m).

Figure 1 shows some characteristic behaviors restored by equations (6) and (7) for single- and two-color laser fields with $I_0 = 5 \times 10^{16}$ W/cm^2 and the relative phase φ set to zero [Fig. 1(a)]. Here, the electron density growth is followed by modulations in time induced by longitudinal plasma oscillations [Fig. 1(b)]. The second row of the same figure [Fig. 1(c,d)] displays the radiated fields in the x- and z-directions. Along x, the THz radiated field exhibits a single-cycled profile - in agreement with the local current scenario [1,2] - while plasma oscillations develop beyond the laser peak along z - in agreement with the "transition-Cerenkov" emission scenario [3]. Both processes produce comparable THz field strengths with two colors. We observe that the two-color pulse amplifies the THz conversion, attached to non-zero low-frequency currents zoomed in Fig. 1(g), whereas longitudinal fields are barely sensitive to the number of colors. The transverse field, promoted by the coupling between the rapid variations of $N_e(t)$ and $E_L(t)$, develops near the laser beam head, while the longitudinal field oscillating with the plasma period extends over long time scales. THz spectra shown in Fig. 1(e,f) evidence the efficiency of the two-color scheme in x and a peak centered around the plasma frequency for the field component along z. The last row demonstrates that the transverse low-frequency currents increase at high pump intensities, even though they relax near the relativistic limit due to the saturation of the ionization process when $Z^* = 8$. In contrast, longitudinal fields develop more and more as the laser intensity increases. For comparison, the red curves illustrate the same quantities for a relative phase of $\pi/2$ between the two colors. This pulse configuration retrieves the well-known property [2,1] following which low-frequency photocurrents induced by tunnel ionization are optimized with a $\pi/2$ phase offset. Originally discovered at moderate intensity, this property still holds in the high intensity range $I_0 > 10^{16}$ W/cm^2.

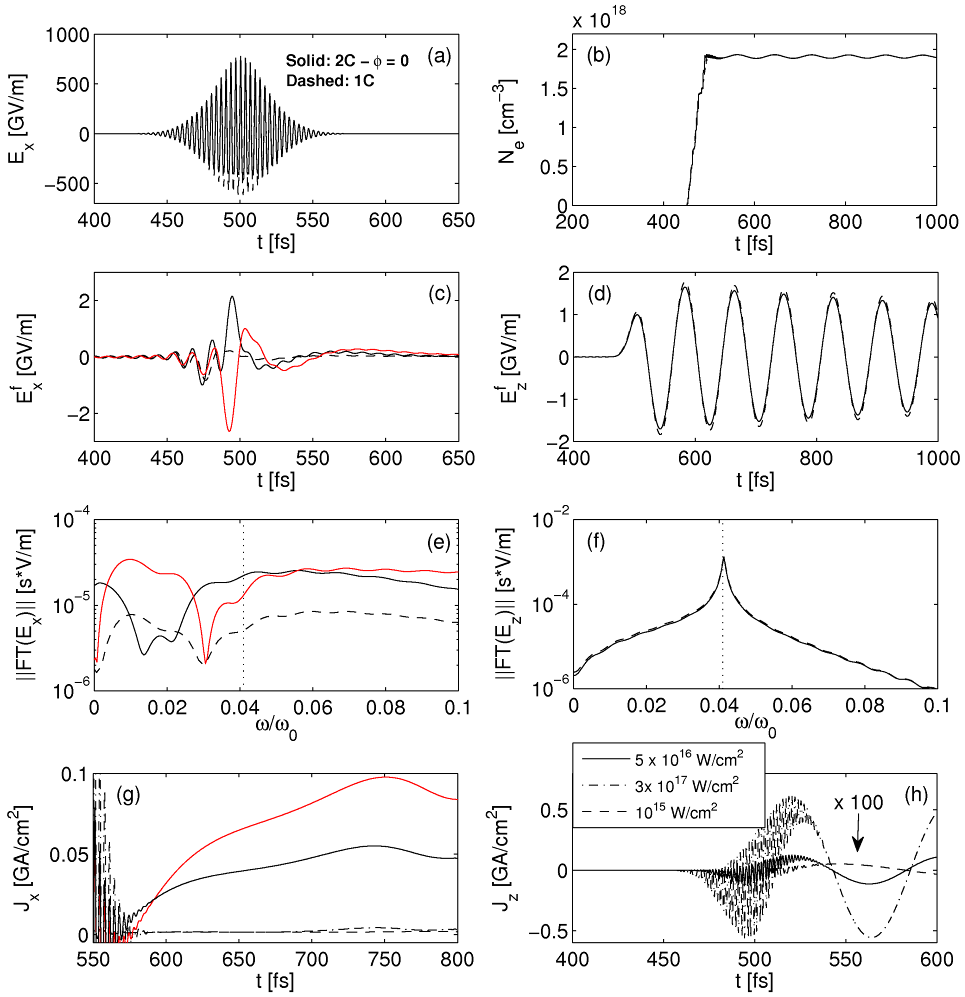

Figure 1: (a) Electric field E_x and (b) electron density versus time for two- (solid curves) and one-color pulses (dashed curves) with 5×10^{16} W/cm^2 pump intensity and zero phase offset ($\varphi = 0$) at $z = 50\,\mu$m. Radiated (c) transverse field E_x and (d) longitudinal field E_z filtered in a frequency window of 90 THz and computed from Eqs (6) and (7). (e) Transverse and (f) longitudinal spectra (FT = Fourier Transform). Vertical dotted lines indicate the position of the plasma frequency (identical for one and two colors). (g) Transverse and (h) longitudinal currents at various laser intensities. The red curves refer to the same plotted quantities when considering a $\pi/2$ initial relative phase between the two colors ($\varphi = \pi/2$).

To confirm the good accuracy of the solutions to Eqs (6) and (7), we compare their profiles with the solutions of the original 1D equation set (17) and (18) for pulses propagating along a 100-μm-long plasma in the z-direction. To do this, we have developed a fully-parallelized 1D Maxwell-fluid code. A second-order splitting algorithm is applied to Eqs (17) and (18) using a source-advection-source scheme. The source is treated with a standard second-order Runge-Kutta scheme; the advection (propagation) is solved by the Richtmyer two-step Lax-Wendroff method [4,5]. To connect vacuum to the plasma zone while avoiding any singularity at their interface, we set fluid velocities (and thus currents) to zero whenever the electron density attains a minimum threshold value [6]. Here, for two-color laser pulses the starting phase offset is $\pi/2$. The comparison is done at $z = 50$ μm inside the plasma range, as the pulse has propagated enough to excite THz emissions.

Figure 2(a-f) show the excellent agreement obtained for $I_0 = 10^{15}$ W/cm^2 between the numerical solutions of the 1D model Eq. (17) and Eq. (18), and of our approximated equations (6) and (7). With a single color, the radiated field polarized along x is smaller than its longitudinal counterpart and longitudinal ponderomotive effects dominate. This tendency is reverted with two colors, the transverse radiated field being ~ 250 times stronger in amplitude. Both transverse and longitudinal spectra appear centered at the plasma frequency. The x-spectrum is broad and covers the entire low-frequency range. By contrast the z-spectrum is highly localized at $\omega = \tilde{\omega}_{\rm pe}$. The red curves show the semi-analytical solutions computed from taking the inverse Fourier transform of Eq. (11) and from Eq. (27) using (30), i.e., when plasma nonlinearities are evaluated under the slowly-varying envelope approximation. This approximation reasonably agrees with the complete 1D solutions, even though it slightly overestimates the longitudinal spectrum. Similar agreements are obtained at higher intensities, as evidenced by Fig. 2(g,h).

In summary, our one-dimensional model given by Eqs (6) and (7) holds over a broad range of intensities and yields a good basis for interpreting 2D PIC simulation results.

References

[1] Babushkin, I., Skupin, S., Husakou, A., Köhler, C., Cabrera-Granado, E., Bergé, L. & Herrmann J. Tailoring terahertz radiation by controlling tunnel photoionization events in gases. *New J. Phys* **13**, 124029 (2011).

[2] Kim, K. Y., Glownia, J. H., Taylor, A. J., & Rodriguez, G. Terahertz emission from ultrafast ionizing air in symmetry-broken laser fields. *Opt. Express* **15**, 4577 (2007).

[3] D'Amico, C., Houard, A., Akturk, S., Liu, Y., Le Bloas, J., Franco, M., Prade, B., Couairon, A., Tikhonchuk, V. T. & Mysyrowicz, A. Forward THz radiation emission by femtosecond filamentation in gases: theory and experiment. *New J. Phys.* **10**, 013015 (2008).

[4] Richtmyer, R. A survey of difference methods for non-steady fluid dynamics. *NCAR Technical Notes* **63-2** (1962).

[5] Zalesak, S. T. The design of flux-corrected transport (FCT) algorithms for structured grids. In "Flux-corrected transport", pp. 29–78, Springer-Verlag (Heidelberg, 2005).

[6] Berezhiani, V. I., Garuchava, D. P., Mikeladze, S. V., Sigua, K. I., Tsintsadze, N. L., Mahajan, S. M., Kishimoto, Y. & Nishikawa K. Fluid-Maxwell simulations of laser pulse dynamics in overdense plasma. *Phys. Plasmas* **12**, 062308 (2005).

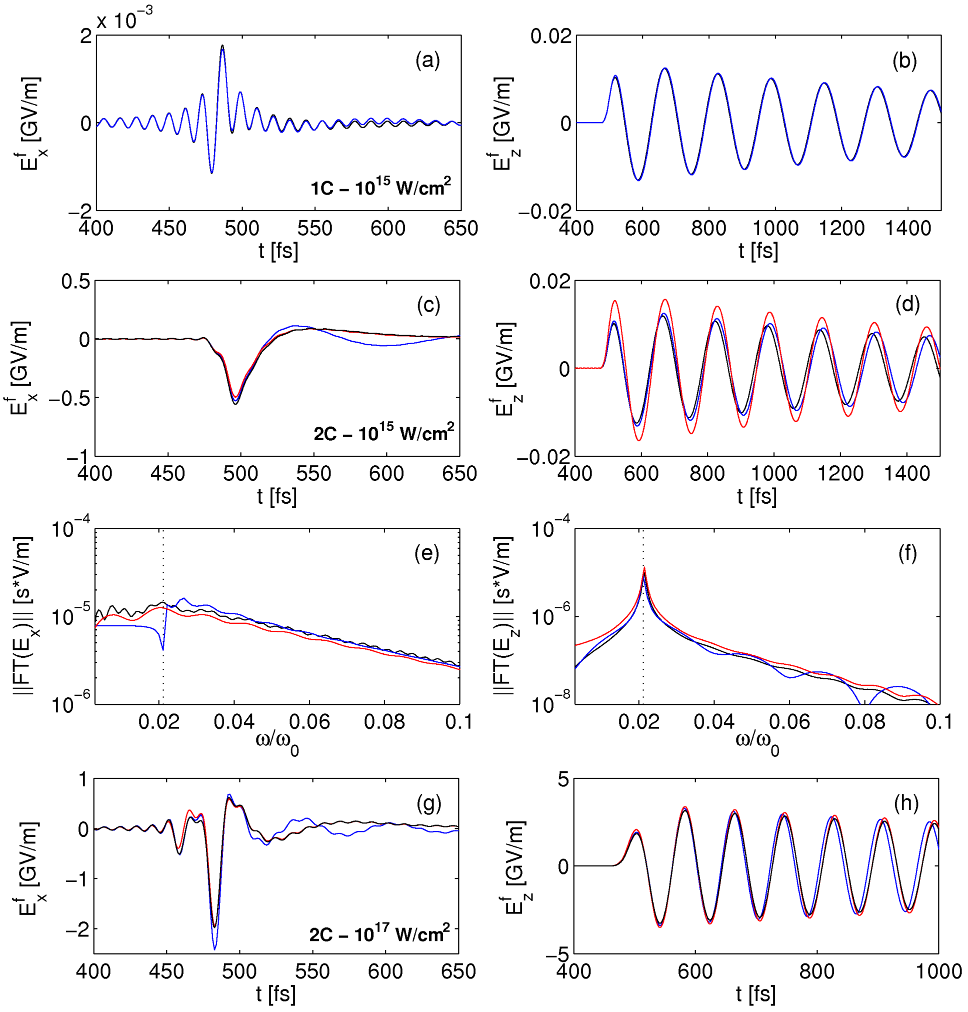

Figure 2: (a,b) Solutions to the 1D model equations (left column) Eq. (17) and (right column) Eq. (18) plotted as blue curves for a pump intensity of 10^{15} W/cm^2 at $z = 50\,\mu$m. The black curves show solutions to Eq. (6) and Eq. (7). (c,d) Solutions for two-color pulses with initial $\pi/2$ dephasing and same intensity. (e,f) Corresponding spectra. The vertical dotted lines indicate the electron plasma frequency. (g,h) Solutions for two colors with a pump intensity of 10^{17} W/cm^2 and same color plotstyle. The red curves illustrate the analytical solutions computed from the inverse Fourier transform of Eq. (11) and from Eq. (27) using the slowly-varying envelopes in Eq. (30).
